# Circadian rhythm of heart rate variability in tropical horses: age-associated autonomic alterations and environmental air pollution effects in an urban field setting

**DOI:** 10.14202/vetworld.2026.2899-2914

**Published:** 2026-07-11

**Authors:** Ashannut Isawirodom, Jakkawat Pongsumpun, Phawita Sangsasithorn, Pongsakorn Petchkaew, Nuttapon Satumay, Kannika Na Lampang, Wanpitak Pongkan, Porrakote Rungsri

**Affiliations:** 1 Faculty of Veterinary Medicine, Chiang Mai University, Chiang Mai, Thailand 50200.; 2 Royal Stable Unit Equine Medical and Rehabilitation Service Centre, Bangkok, Thailand 10400.; 3 Department of Veterinary Medicine, Faculty of Veterinary Medicine, Chiang Mai University, Chiang Mai, Thailand 50200.

**Keywords:** aging, air pollution, autonomic nervous system, circadian rhythm, heart rate variability, horse, particulate matter 2.5, tropical climate

## Abstract

**Background and Aim:**

Heart rate variability (HRV) reflects autonomic nervous system activity and is influenced by age, environmental conditions, and circadian rhythms. Information regarding these interactions in horses maintained under tropical conditions remains limited. This study aimed to characterize 24-h heart rate (HR) and HRV patterns in horses raised under tropical conditions and to investigate the effects of aging and environmental factors, including air pollution, on autonomic regulation.

**Materials and Methods:**

Fifteen clinically healthy horses aged 4-20 years were allocated to three age groups: 4-7 years, 8-14 years, and 15-20 years (n = 5 per group). Continuous 24-h recordings of HR and HRV were obtained using a Polar H10 sensor. Time-domain and frequency-domain HRV indices were analyzed using Kubios Scientific software. Simultaneously, environmental parameters, including temperature, humidity, feels-like temperature, light intensity, air quality index (AQI), and particulate matter ≤2.5 μm (PM2.5), were continuously monitored under field conditions in Bangkok, Thailand. Circadian variations, age-associated differences, and correlations between HRV and environmental variables were evaluated.

**Results:**

Only HR and normal-to-normal intervals differed significantly between daytime and nighttime, whereas other HRV indices exhibited limited circadian variation, indicating attenuated nocturnal parasympathetic predominance. Older horses (15–20 years) demonstrated significantly lower parasympathetic-related indices, including root mean square of successive differences (59.44 ± 7.25 ms) and high-frequency (HF) power (805.60 ± 213.37 ms²), compared with younger groups (p < 0.05). Total power was reduced, whereas the low-frequency (LF)/HF ratio increased with age, suggesting diminished autonomic flexibility and sympathetic predominance. Air pollution variables showed significant associations with autonomic imbalance. Both AQI and PM2.5 were negatively correlated with HRV indices, including root mean square of successive differences, percentage of normal-to-normal intervals differing by >100 ms, and HF power, while showing positive correlations with the LF/HF ratio (p < 0.001).

**Conclusion:**

Tropical horses exhibited blunted circadian autonomic modulation and age-related reductions in parasympathetic activity. Furthermore, elevated PM2.5 and AQI levels were associated with impaired autonomic balance, providing the first evidence linking ambient air pollution with resting HRV alterations in horses. These findings emphasize the importance of age-specific management and environmental monitoring to promote equine welfare in tropical urban environments.

## INTRODUCTION

Circadian rhythm refers to a daily cycle that regulates physiological processes in most living organisms over a 24-hour period [1, 2]. This rhythm is controlled by the suprachiasmatic nucleus, located in the hypothalamus, which responds to both internal and external factors, particularly light intensity, to maintain physiological homeostasis [3–5]. Among cardiovascular parameters, heart rate variability (HRV) is a well-established non-invasive marker of autonomic nervous system activity, reflecting the dynamic balance between sympathetic and parasympathetic modulation [3, 6]. HRV exhibits circadian variation, providing insight into autonomic adaptability under physiological and environmental influences [7–9].

In horses, circadian fluctuations of heart rate (HR) and HRV have been reported primarily under temperate conditions characterized by pronounced day–night temperature gradients and controlled housing systems [10–13]. However, information regarding horses maintained under tropical environments remains limited. Tropical conditions present distinct challenges, including persistently high ambient temperature, high humidity, limited nocturnal cooling, and environmental stressors such as air pollution, particularly in urban settings [14, 15]. In addition, biological disturbances, including insect activity during nighttime, may interfere with rest and recovery, potentially affecting autonomic regulation [16]. These factors may alter circadian autonomic patterns and attenuate the nocturnal parasympathetic predominance typically observed in temperate climates [10, 17]. Aging is also recognized as an important factor influencing HRV [10, 18], with advancing age generally associated with reduced autonomic flexibility and diminished parasympathetic activity.

Although previous studies have characterized circadian changes in HRV and age-associated autonomic alterations in horses, most investigations have been conducted under temperate climatic conditions and have focused on either circadian variation or aging independently [10–13, 17, 18]. Consequently, the combined influence of tropical environmental conditions, age, and circadian rhythm on equine autonomic regulation remains insufficiently understood. Furthermore, previous studies have generally relied on controlled experimental settings and have rarely incorporated continuous monitoring of environmental variables under real-world field conditions. In particular, the potential impact of urban air pollution on resting autonomic function in horses has received little attention, despite growing concerns about environmental quality and equine welfare. Therefore, important knowledge gaps remain regarding how tropical environmental stressors interact with age-related changes and circadian regulation to influence cardiac autonomic activity in horses.

Therefore, this study aimed to comprehensively investigate the circadian characteristics of HR and HRV in horses maintained under tropical environmental conditions. Specifically, the study sought to characterize 24-hour circadian patterns of HR and HRV, compare autonomic regulation between daytime and nighttime, evaluate age-associated alterations in cardiac autonomic function, and examine relationships between environmental factors and HRV parameters. We hypothesized that tropical environmental conditions would attenuate nocturnal parasympathetic predominance, aging would reduce overall HRV, and environmental stressors, particularly heat and air pollution, would shift autonomic balance toward sympathetic predominance.

To the best of our knowledge, this is the first study to simultaneously investigate 24-hour circadian HRV, age-related autonomic alterations, and real-time associations with tropical environmental factors, including air pollution parameters, in a standardized field setting. The findings provide novel insights into how urban tropical environments influence autonomic aging and equine welfare, factors that have not previously been quantified in equine physiology.

## MATERIALS AND METHODS

### Ethical approval

The study protocol was reviewed and approved by the Laboratory Animal Center, Chiang Mai University, Thailand, which serves as the institution's Institutional Animal Care and Use Committee (IACUC) (approval code: AG001/2567). Written informed consent was obtained from the horse owners or authorized caretakers before enrollment. Only clinically healthy horses with owner authorization were included. All procedures were non-invasive and involved external 24-h HR and HRV monitoring using a chest strap sensor under routine management conditions. No experimental treatment, restraint beyond routine handling, invasive sampling, or procedure likely to cause pain or distress was performed. Horses were housed and managed according to their normal stable routine, with ad libitum access to water and regular feeding, grooming, and light activity schedules. During the monitoring period, animals were continuously observed by trained personnel for signs of discomfort, distress, abnormal behavior, or device-related irritation, and the monitoring device could be removed immediately if any welfare concern arose. All efforts were made to minimize disturbance and ensure animal welfare throughout the study.

### Study period and location

The study was conducted in late January 2025 in Bangkok, Thailand, during the winter season in a tropical savanna climate. During the study period, ambient temperature ranged from 23°C to 33°C, while relative humidity ranged from 50% to 85%. These environmental conditions provided a representative tropical winter setting for investigating circadian autonomic regulation and the influence of environmental variables in horses maintained under field conditions.

### Study design

This study was designed as an observational cross-sectional field study conducted under tropical environ-mental conditions. Horses were categorized into three age groups, and continuous 24-hour monitoring of HRV and environmental parameters was performed. Data collection was conducted over multiple days, with one horse from each age group monitored simultaneously during each recording session (three horses/day).

The protocol combined non-invasive 24-hour recordings using a Polar H10 HR sensor (Polar Electro, Kempele, Finland) in open-air tropical stalls under standardized management conditions with synchronized stall-level environmental monitoring using a WH2900C weather station (Shenzhen Fine Offset Electronics Co., Ltd., Shenzhen, China). This real-world approach provided an ecologically valid assessment of autonomic regulation in relation to both management-related and environmental influences under tropical winter conditions.

### Horses

A total of 18 horses were initially enrolled in the study. Three animals were excluded because of excessive artifacts or poor compliance with the monitoring device. Consequently, only datasets meeting the predefined quality criteria were included in the final analysis, resulting in a study population of 15 horses.

The horses ranged from 4 to 20 years of age and comprised 12 geldings, two mares, and one stallion. Breed composition included eight Warmblood horses, six Warmblood-cross horses, and one Anglo-Arabian horse. The mean age and body weight were 12.06 ± 5.21 years and 530.13 ± 62.35 kg, respectively, with body weights ranging from 445 to 620 kg. Body condition scores ranged from 3 to 4 according to the scoring system described by Carroll and Huntington [19]. All horses were used for riding-school activities and were maintained under comparable management conditions, with no history of intensive athletic training.

The inclusion criteria consisted of clinically healthy horses with normal findings on physical examination, auscultation, electrocardiography, and echocardiography and without a history of systemic illness or administration of medications during the preceding two months. Horses with cardiac abnormalities or active medical conditions were excluded from the study.

**Eligible horses were allocated to three age groups:** Group 1 (4–7 years), Group 2 (8–14 years), and Group 3 (15–20 years). Detailed characteristics of the study groups are presented in Table 1.

**Table 1:** Characteristics of the study groups. Horses were categorized into Group 1 (4–7 years), Group 2 (8–14 years), and Group 3 (15–20 years). Age and body weight are presented as mean ± standard deviation (SD).

**Characteristic**	**Category**	**Group 1 (4–7 years)**	**Group 2 (8–14 years)**	**Group 3 (15–20 years)**
**Sex**	Stallion (n)	1	0	0
	Gelding (n)	4	4	4
	Mare (n)	0	1	1
**Breed**	Warmblood (n)	2	1	5
	Warmblood-cross (n)	3	3	0
	Anglo-Arabian (n)	0	1	0
**Age (years)**		5.5 ± 1.10	11.8 ± 1.92	17.4 ± 1.82
**Body weight (kg)**		463.60 ± 12.97	575.40 ± 37.31	551.40 ± 58.20

Horses were allocated to age groups based on predefined age ranges, and no randomization was performed. During each recording session, one horse from each age group was monitored simultaneously under identical environmental conditions. Efforts were made to select animals with comparable body condition scores; however, some variation in body weight and breed distribution among groups remained because of population availability.

### General physical examination

Before enrollment, all horses underwent a comprehensive physical examination, including assessment of vital signs (HR, respiratory rate, and rectal temperature), cardiac and pulmonary auscultation, evaluation of mucous membrane color and capillary refill time, and gastrointestinal auscultation. All animals were clinically healthy and had no history of medication administration during the previous two months. Furthermore, no horse exhibited signs of illness or was undergoing treatment at the time of the study.

### HR and HRV monitoring

**Figure 1:**
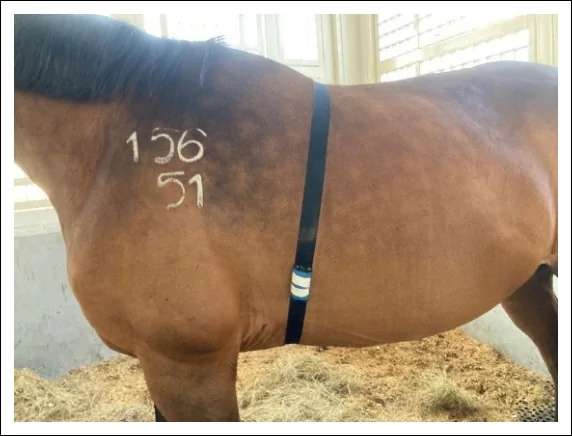
Placement of the heart rate sensor on the horse. The sensor was fastened with a trotter strap behind the withers, with the unit aligned vertically between the shoulder and elbow.

HR and normal-to-normal intervals (NN) were continuously recorded for 24 hours in all horses using a Polar H10 HR sensor (Polar Electro) with a sampling frequency of 1,000 Hz. Before sensor placement, horses were thoroughly groomed to remove debris from the electrode contact area. The sensor was secured with a non-invasive, equine-specific chest strap (Polar Electro), pre-wetted with water to optimize electrical conductivity between the skin and the electrodes, according to the manufacturer's instructions. Following skin preparation, the chest strap was positioned behind the withers, with the sensor unit aligned vertically between the shoulder and elbow (Figure 1).

Data acquisition began at 06:00 a.m. and continued uninterrupted for 24 hours. Sensor connectivity and data acquisition were managed using the Kubios HRV mobile application (version 1.7.12; Kubios Oy, Kuopio, Finland), which ensured synchronization and recording integrity. Signal quality and sensor position were regularly checked throughout the recording period to minimize motion-related artifacts.

Throughout the monitoring period, horses were housed individually in open-air stalls equipped with ceiling- or wall-mounted fans to facilitate air circulation. Horses maintained visual and auditory contact with neighboring animals and had ad libitum access to fresh drinking water.

The horses followed a standardized daily routine that included scheduled feeding, grooming, and light activity. Concentrate and roughage were provided at fixed times, whereas grooming and hand walking were performed during the daytime. Human interaction was minimized at night. The daily activity schedule is summarized in Table 2.

**Table 2:** Daily activity schedule of horses during resting days.

**Time**	**Activity**
04:00	Concentrate feeding
06:00	Heart rate sensor attachment
07:00	Grooming
09:00	Roughage feeding
11:00	Concentrate feeding
13:00	Body temperature monitoring
15:30	Grooming
16:00	Hand walking
17:00	Return to stall
20:00	Concentrate feeding
21:00	Roughage feeding

### HRV data analysis

The 24-h recording period was divided into eight-time intervals to evaluate circadian patterns of HR and HRV. For day–night comparisons, recordings were categorized into daytime (06:00–18:00) and nighttime (18:00–06:00). HRV parameters were also analyzed over the entire 24-hour period.

Raw inter-beat interval data were analyzed using Kubios Scientific HRV software (version 4.1.2.1; Kubios Oy, Kuopio, Finland). All RR interval tachograms were visually inspected for irregularities. Although electrocardio-graphy was not performed to confirm physiological arrhythmias, such as second-degree atrioventricular block, manual inspection was rigorously performed to minimize their influence on HRV analysis.

The threshold-based artifact correction algorithm implemented in Kubios Scientific HRV software was applied using a medium correction level (threshold = 0.25 s). Recordings were accepted only when the proportion of corrected beats remained below 5%, ensuring that >95% of RR intervals were suitable for analysis. Segments with excessive noise or poor signal quality were excluded. Missing or ectopic beats were corrected using cubic spline interpolation. To reduce low-frequency (LF) non-stationarity, detrending was performed using the smoothness priors method (λ = 500, cutoff frequency = 0.035 Hz). Each 24-hour recording underwent complete visual inspection to ensure overall signal integrity. Frequency-domain variables were automatically calculated using consecutive 5-minute epochs.

Time-domain variables included NN, standard deviation of NN intervals (SDNN), root mean square of successive differences (RMSSD), standard deviation of the averages of NN intervals for each 5-minute segment (SDANN), and the percentage of NN intervals differing by >100 ms (pNN100). In the present study, pNN100 was used instead of pNN50 as a species-specific adaptation because horses characteristically exhibit lower HR and longer RR intervals [20].

Fast Fourier transformation was used to convert NN intervals into frequency components. Frequency-domain analysis was performed using predefined frequency bands. The very low-frequency (VLF) range was defined as 0.001–0.01 Hz, the LF range as 0.01–0.12 Hz, and the high-frequency (HF) range as 0.12–0.6 Hz. The HF range was selected based on the respiratory frequency of the study population (0.18 ± 0.02 Hz) and previously reported equine HRV frequency ranges [21–23]. Variables analyzed included VLF power, LF power, HF power, total power, and the LF/HF ratio.

### Environmental parameter monitoring

Environmental variables were continuously monitored over 24-hour periods to evaluate their associations with HRV parameters. Variables included ambient temperature, relative humidity, feels-like temperature, light intensity, air quality index (AQI), and particulate matter ≤2.5 μm (PM2.5). Feels-like temperature represented a composite index incorporating temperature, humidity, and wind speed. Both temperature variables were retained to capture ambient conditions and perceived thermal load. Because of their inherent correlation, they were not simultaneously incorporated into the same model.

Environmental data were collected using a factory-calibrated weather station (WH2900C; Shenzhen Fine Offset Electronics Co., Ltd.). The station was installed at a height of 1.8 m within the stable area to reflect the immediate environment of the horses. Environmental data were transmitted and stored in real time via the Ecowitt cloud platform (Ecowitt, Shenzhen, China), enabling synchronization with physiological recordings. Both the weather station and mobile devices used for HRV acquisition were synchronized to the same network time source. No significant data loss occurred during the study.

### Statistical analysis

An *a priori* sample size calculation was performed using G*Power software (version 3.1.9.4; Heinrich Heine University Düsseldorf, Düsseldorf, Germany). RMSSD was selected as the primary outcome because of its sensitivity to parasympathetic modulation and relevance to age-related changes. Based on a previous equine study, a one-way analysis of variance model with three groups (α = 0.05, power = 0.80, effect size *f* = 1.23) indicated that 12 horses were required. Therefore, the inclusion of 15 horses was considered adequate.

Data are expressed as mean ± SD. Normality was evaluated using the Shapiro-Wilk test and quantile-quantile plots, whereas homogeneity of variance was assessed using Levene's test. No data transformation was required.

A mixed-model analysis of variance was used to evaluate the effects of time (within-subject) and group (between-subject), with horse identification included as a random effect to account for repeated measurements. Effect sizes for group, time, and interaction terms were reported using generalized eta-squared. When significant interaction effects were detected, simple main effects were explored. Post hoc comparisons were performed using pairwise *t*-tests with Holm correction, and effect sizes were expressed as Cohen's *d*.

Correlations between HRV parameters and environmental variables were assessed using Spearman's rank correlation coefficient. Environmental variables were analyzed individually in relation to HRV parameters and were not included simultaneously in multivariable models; therefore, multicollinearity was not considered a concern. All statistical analyses were performed using R software (version 4.5.0; R Foundation for Statistical Computing, Vienna, Austria), and statistical significance was set at p < 0.05**.**

## RESULTS

**Circadian HR and HRV patterns showed evening sympathetic predominance and blunted nocturnal para** **-** **sympathetic modulation**

**Figure 2:**
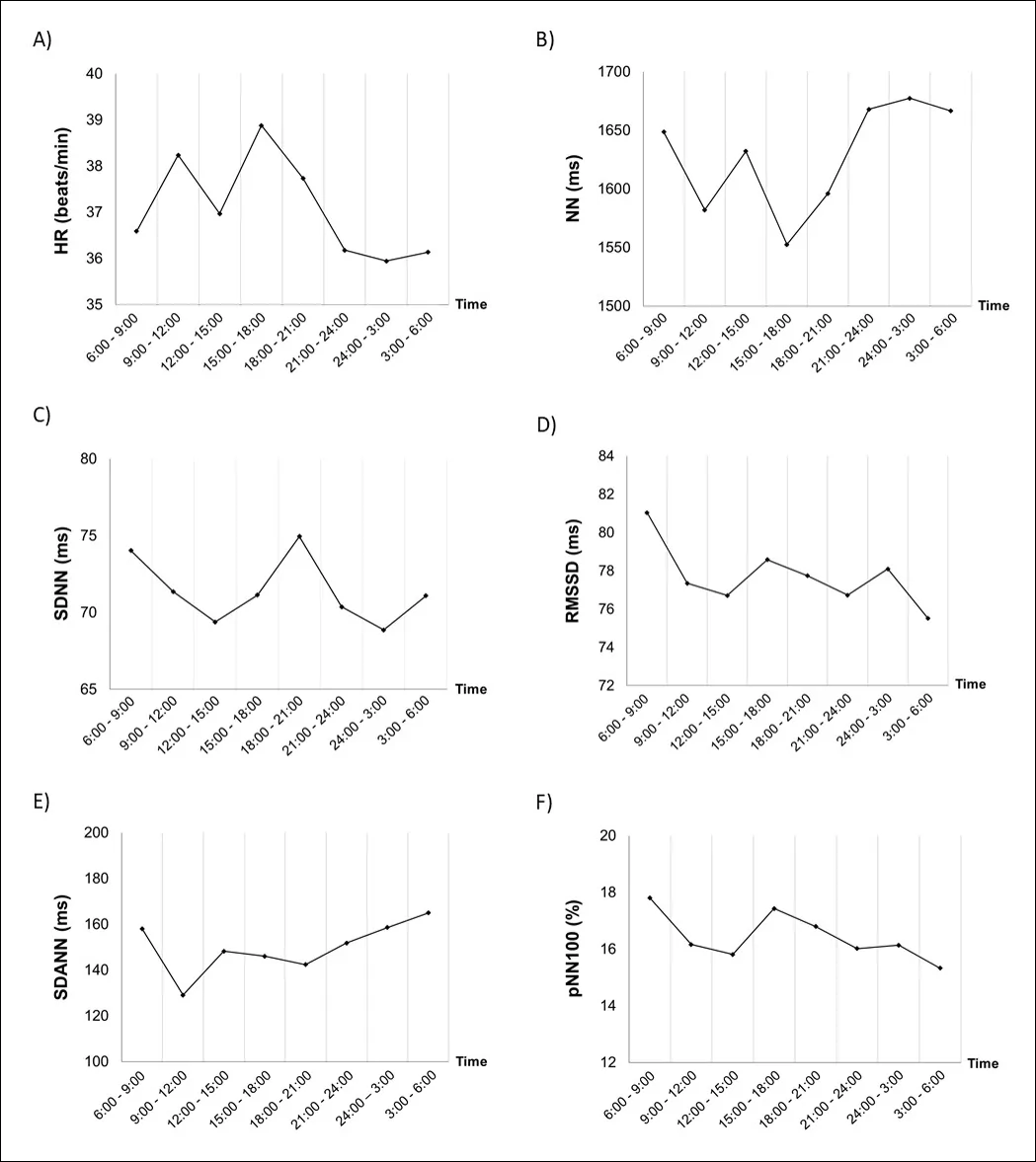
Circadian rhythm of HR and time-domain HRV parameters, including NN, SDNN, RMSSD, SDANN, and pNN100. Data represent mean values across eight time segments throughout the 24-hour period.

Across the full 24-hour period, all 15 horses demonstrated consistent patterns of cardiac autonomic regulation. HR showed a clear diurnal pattern, with the highest value during daytime hours (38.88 ± 3.13 beats/min at 15:00–18:00) and the lowest value during nighttime (35.94 ± 2.57 beats/min at 24:00–03:00). Conversely, NN exhibited an inverse trend, as shown in Figures 2A and B.

Time-domain HRV parameters demonstrated relatively stable patterns across the 24-hour period (Figures 2C–F). SDNN values were lowest during 24:00–03:00 (68.87 ± 9.76) and highest during 18:00–21:00 (74.94 ± 8.59), whereas RMSSD values remained relatively consistent, with a peak at 06:00–09:00 (81.04 ± 20.00) and the lowest value at 03:00–06:00 (75.52 ± 15.55). SDANN decreased in the late morning, followed by a gradual increase at night. pNN100 showed a pattern similar to RMSSD. The overall trends of these parameters across the 24-hour cycle are illustrated in Figure 2.

**Figure 3:**
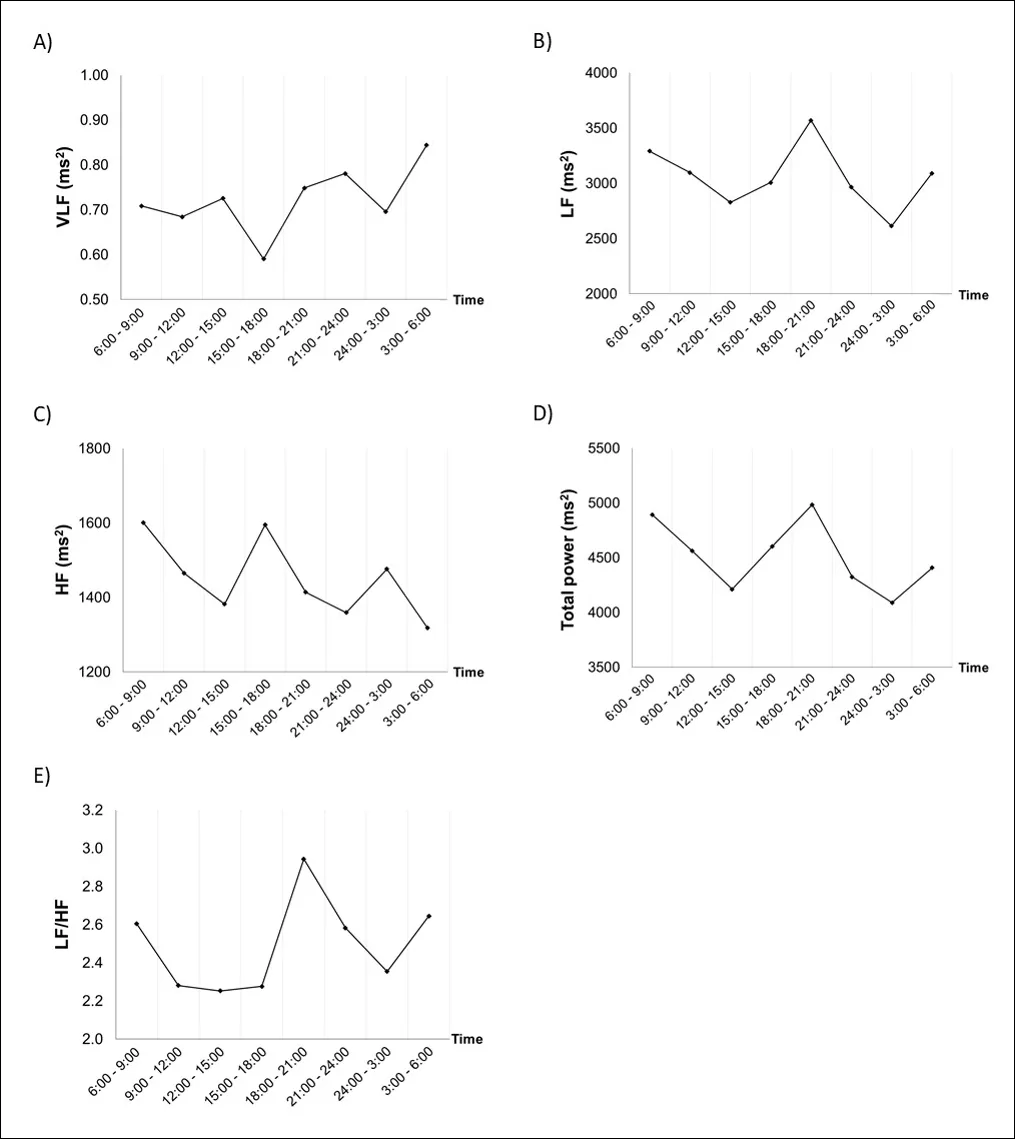
Circadian rhythm of frequency-domain HRV parameters, including VLF power, LF power, HF power, total power, and LF/HF ratio. Data represent mean values across eight time segments throughout the 24-hour period.

Frequency-domain HRV parameters showed modest variation across the 24-hour period. VLF remained relatively stable, with only minor fluctuations throughout the day. LF power and total power peaked during the early evening (18:00–21:00), whereas lower values were observed during late nighttime. HF power exhibited relatively small changes across time intervals without a clear nocturnal elevation. The LF/HF ratio was highest during the evening (2.94 ± 1.31 at 18:00–21:00) and lowest during midday, indicating a transient shift toward sympathetic predominance during this period. The trends of each frequency-domain parameter are shown in Figure 3.

Overall, frequency-domain patterns were consistent with time-domain findings. RMSSD, pNN100, and HF showed similar temporal trends, whereas mixed autonomic markers (SDNN and LF) and long-term variability indices (SDANN and VLF) demonstrated comparable fluctuations. However, only modest circadian variation was observed, with a transient evening increase in sympathetic predominance and no clear nocturnal parasympathetic predominance. Detailed mean ± SD values for all parameters across time intervals are provided in Supplementary Table S1.

### HR decreased at night, whereas autonomic balance showed minimal day–night variation

**Figure 4:**
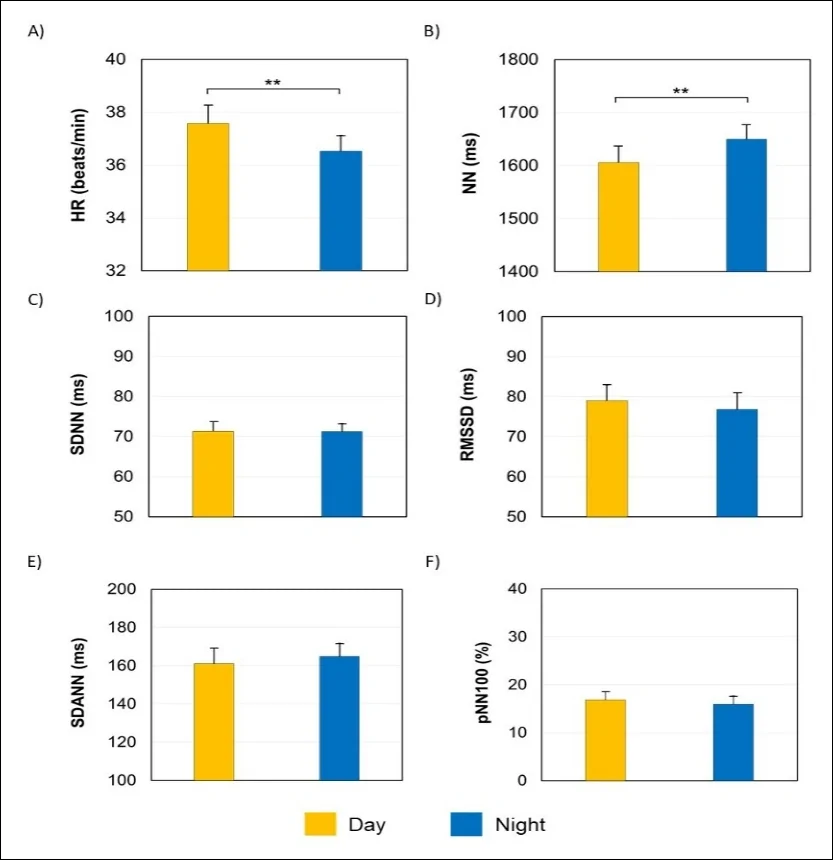
Comparison of HR and time-domain HRV parameters between daytime and nighttime. Parameters include NN, SDNN, RMSSD, SDANN, and pNN100. Error bars indicate the standard error of the mean, and significance levels between daytime and nighttime are indicated by **p < 0.01 versus daytime.

Overall, time-domain indices remained largely stable between daytime (06:00–18:00) and nighttime (18:00–06:00), with significant differences observed only for HR and NN. Daytime HR (37.57 ± 2.80 beats/min) was higher than nighttime HR (36.52 ± 2.38 beats/min, p = 0.004), consistent with a small but significant time effect in the mixed-model analysis of variance (p < 0.001, generalized eta-squared = 0.035). Similarly, NN was shorter during daytime (1605.77 ± 125.06 ms) than during nighttime (1649.58 ± 110.75 ms, p = 0.005), consistent with the overall time effect (p = 0.001, generalized eta-squared = 0.030). In contrast, other HRV parameters (SDNN, RMSSD, SDANN, and pNN100) showed no significant day–night differences (all p > 0.05), indicating attenuated circadian modulation. These comparisons are illustrated in Figure 4.

**Figure 5:**
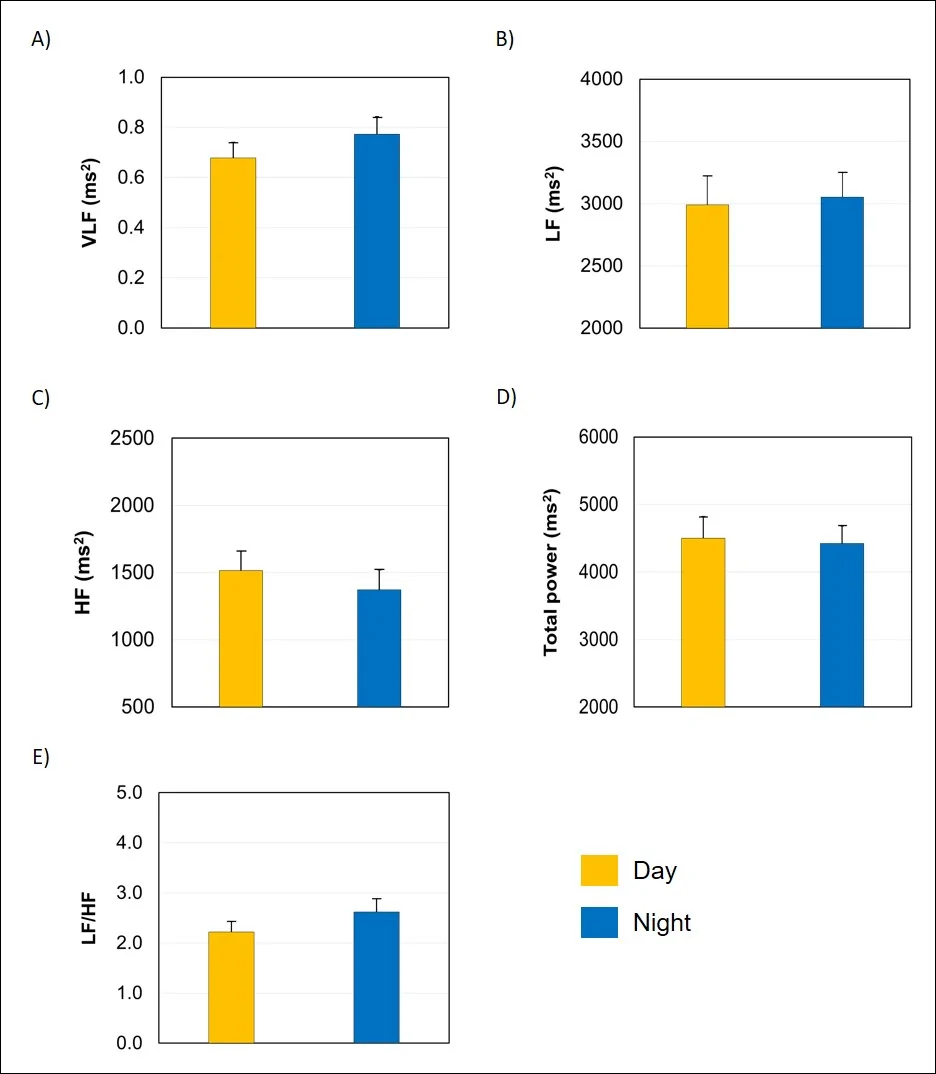
Comparison of frequency-domain HRV parameters between daytime and nighttime. Parameters include VLF power, LF power, HF power, total power, and LF/HF ratio. Error bars indicate the standard error of the mean.

In the frequency-domain, no parameters differed significantly between daytime and nighttime (all p > 0.05). VLF and LF power were slightly lower during daytime than during nighttime. HF power was higher during the daytime than at night, but the difference was not significant. Total spectral power remained comparable between the two periods. The LF/HF ratio increased non-significantly from 2.22 ± 0.86 during daytime to 2.62 ± 1.08 during nighttime. The frequency-domain comparison between daytime and nighttime is illustrated in Figure 5.

Compared with temperate-climate studies that reported pronounced nocturnal parasympathetic elevation, horses in this tropical setting showed only modest HR reduction with blunted changes in RMSSD, pNN100, and HF power, indicating attenuated circadian modulation under these conditions. Detailed mean ± SD values for all parameters during daytime and nighttime are provided in Supplementary Table S2.

### Aging horses showed impaired HRV compared with younger horses

To examine age-related differences, HR and HRV parameters were averaged over the 24-hour recording period and compared among groups. Mixed-model analysis of variance revealed significantly large group effects for multiple HRV parameters, including SDNN, RMSSD, pNN100, HF, total power, and LF/HF ratio (all p < 0.05), indicating age-related differences in autonomic regulation. No significant group × time interactions were observed for any parameter.

**Figure 6:**
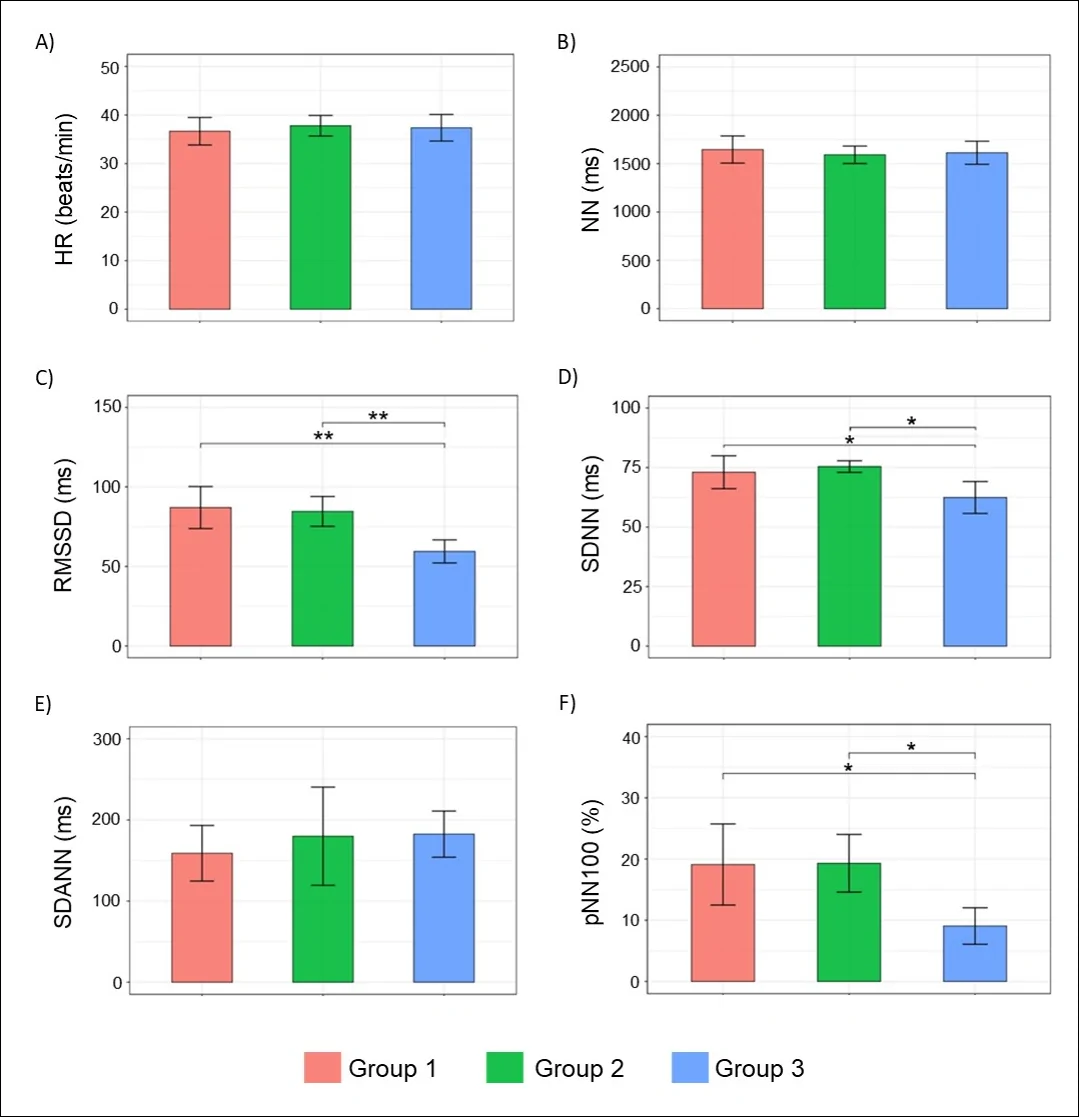
Comparison of HR and time-domain HRV parameters among the three age groups: Group 1 (4–7 years), Group 2 (8–14 years), and Group 3 (15–20 years). Parameters include NN, SDNN, RMSSD, SDANN, and pNN100. Error bars indicate the standard error of the mean, and significance levels between groups are indicated by *p < 0.05 and **p < 0.01.

Time-domain analysis showed minimal differences in HR and NN among groups. In contrast, short-term variability indices demonstrated clear age-related effects. SDNN was significantly lower in Group 3 than in Group 1 (p = 0.025) and Group 2 (p = 0.011), whereas Groups 1 and 2 did not differ. A similar pattern was observed for RMSSD, with Group 3 showing significantly lower values than Group 1 (p = 0.003) and Group 2 (p = 0.004), with no difference between Groups 1 and 2. pNN100 showed the clearest separation, with Group 3 having significantly lower values than both Group 1 and Group 2 (both p = 0.021), whereas Groups 1 and 2 did not differ. In contrast, long-term variability, as assessed by SDANN, showed no significant differences among groups. These comparisons are illustrated in Figure 6.

**Figure 7:**
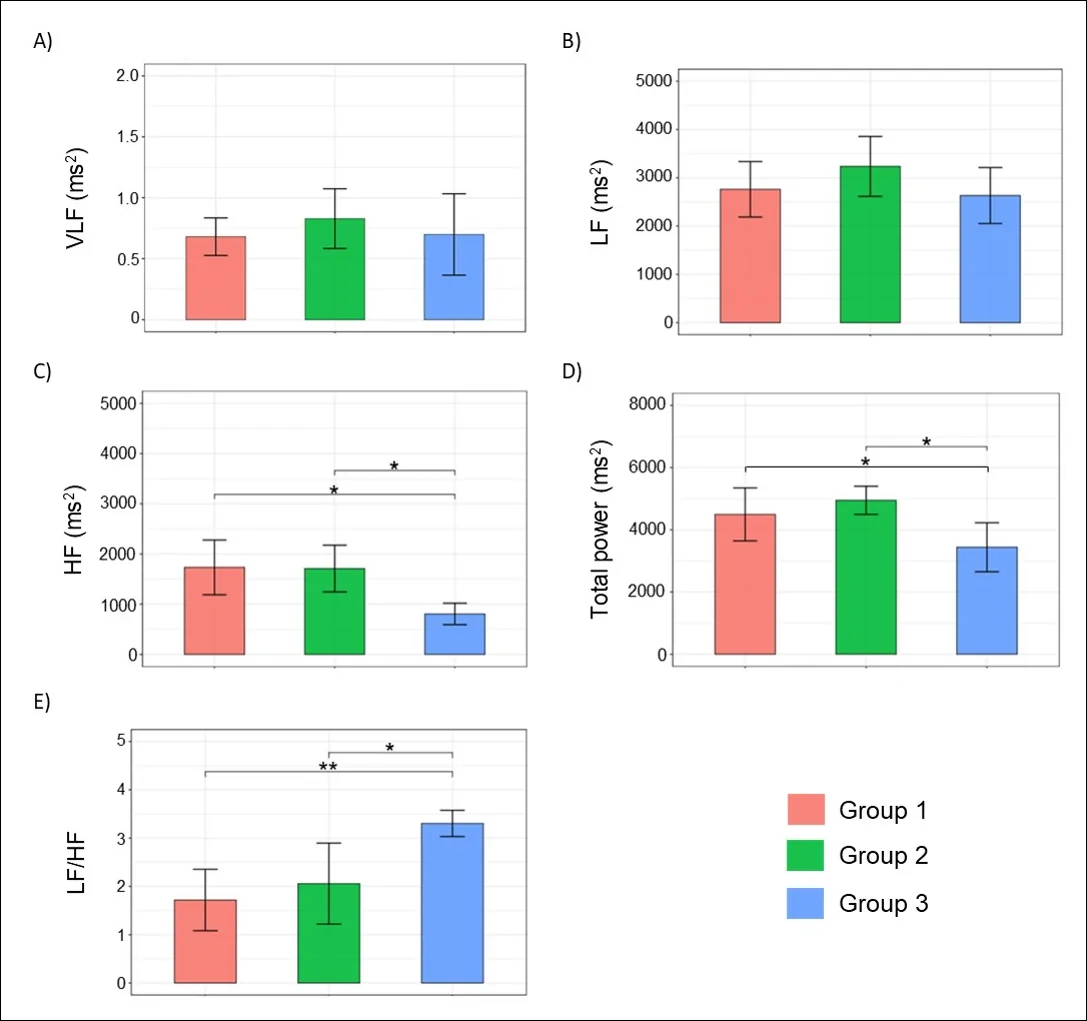
Comparison of frequency-domain HRV parameters among the three age groups: Group 1 (4–7 years), Group 2 (8–14 years), and Group 3 (15–20 years). Parameters include VLF power, LF power, HF power, total power, and LF/HF ratio. Error bars indicate the standard error of the mean, and significance levels between groups are indicated by *p < 0.05 and **p < 0.01.

When frequency-domain HRV parameters were compared among groups, VLF power remained relatively similar across all age groups, whereas LF power showed only modest differences. Conversely, HF power showed the clearest group separation, with Group 3 showing significantly lower values than Groups 1 and 2 (both p = 0.0159). Total spectral power followed a similar pattern, with Group 3 showing significantly lower values than Group 1 (p = 0.039) and Group 2 (p = 0.018). The LF/HF ratio increased progressively with age, with Group 3 showing significantly higher values than Group 1 (p = 0.005) and Group 2 (p = 0.017). These findings demonstrate reduced parasympathetic activity and a shift toward sympathetic predominance in older horses. The across-group comparisons are illustrated in Figure 7.

These findings indicate that older horses (15–20 years) displayed markedly reduced short-term vagal activity and elevated LF/HF ratios, extending previous age-related findings by demonstrating that impaired HRV occurs even at rest under tropical conditions. Detailed numerical values are provided in Supplementary Table S3.

### Environmental parameters were associated with HRV parameters

Feels-like temperature and light intensity were significantly higher during daytime, whereas humidity increased at night. PM2.5 and AQI showed relatively minor day–night differences but remained highly variable. Environmental values during daytime, nighttime, and the 24-hour period are shown in Table 3.

**Table 3:** Day-night variation in environmental parameters is presented as mean ± SD with statistical comparison.

**Environmental parameter**	**Day** **time (mean ± SD)**	**Night** **time (mean ± SD)**	**p-value**
Temperature (°C)	28.65 ± 1.45	26.43 ± 1.09	<0.001
Humidity (%)	54.08 ± 2.86	61.85 ± 5.04	<0.001
Feels-like temperature (°C)	29.46 ± 2.04	27.09 ± 1.73	0.008
Light intensity (lux)	6214.55 ± 751.84	5.09 ± 5.47	<0.001
AQI	134.33 ± 30.87	133.04 ± 25.08	0.99
PM2.5 (µg/m³)	54.12 ± 17.66	52.03 ± 14.94	0.95

Correlation analysis showed no significant associations between temperature or feels-like temperature and any HRV parameter. However, humidity was significantly negatively correlated with HR (r = −0.230, p < 0.001) and positively correlated with NN (r = 0.230, p < 0.001). Light intensity showed a significant positive correlation with HR (r = 0.166, p < 0.001) and negative correlations with NN (r = −0.166, p < 0.001) and SDANN (r = −0.111, p = 0.029).

Both AQI and PM2.5 demonstrated consistent associations with cardiorespiratory parameters. Specifically, both variables were negatively correlated with HR (AQI: r = −0.318, p < 0.001; PM2.5: r = −0.320, p = 0.002) and positively correlated with NN (AQI: r = 0.318, p < 0.001; PM2.5: r = 0.320, p = 0.003). In addition, the respiratory cycle was reduced with increasing air pollution levels (AQI: r = −0.323, p < 0.001; PM2.5: r = −0.332, p < 0.001). AQI and PM2.5 were also significantly negatively correlated with parasympathetic-related indices, including RMSSD (AQI: r = −0.239, p < 0.001; PM2.5: r = −0.235, p < 0.001), pNN100 (AQI: r = −0.238, p < 0.001; PM2.5: r = −0.231, p < 0.001), and HF power (AQI: r = −0.300, p < 0.001; PM2.5: r = −0.295, p < 0.001).

Conversely, both AQI and PM2.5 showed significant positive correlations with LF/HF ratio (r = 0.318, p < 0.001 for both), indicating a shift toward sympathetic predominance. Total power was also negatively associated with AQI (r = −0.323, p < 0.001) and PM2.5 (r = −0.332, p < 0.001).

**Figure 8:**
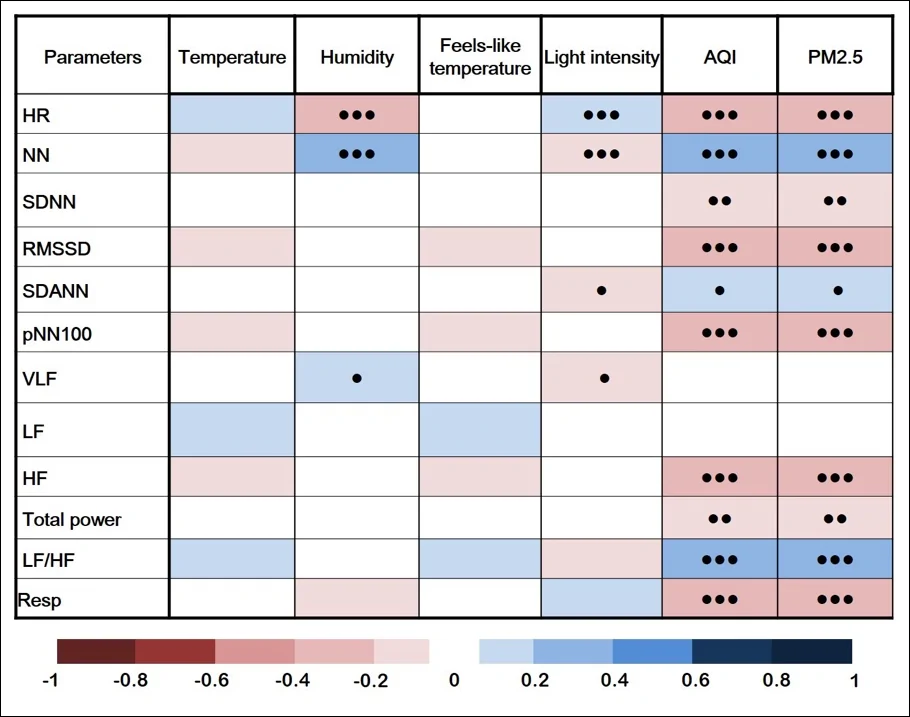
Correlation between environmental parameters and HRV indices. Environmental parameters include temperature, humidity, feels-like temperature, light intensity, AQI, and PM2.5. HRV parameters include HR, NN, SDNN, RMSSD, SDANN, pNN100, VLF power, LF power, HF power, total power, and LF/HF ratio. Respiratory cycle is included. Statistical significance levels: ● = p < 0.05, ●● = p < 0.01, and ●●● = p < 0.001.

Positive correlations between PM2.5/AQI and sympathetic predominance, reflected by LF/HF ratio, provide direct evidence linking ambient air pollution to impaired autonomic function in horses. Correlations between environmental variables and HRV parameters, including respiratory cycle, are summarized in Figure 8 and Supplementary Table S4.

## DISCUSSION

### Overview of key findings

This study provides original evidence that tropical environmental conditions attenuate the expected circadian HRV pattern, with only modest nocturnal HR slowing and no clear parasympathetic surge. Furthermore, it links real-time air quality metrics, particularly PM2.5 and AQI, to altered autonomic regulation, characterized by reduced HRV, concurrent decreases in HR, and respiratory cycle changes. These findings suggest a complex and potentially species-specific response that has not previously been described in horses under field conditions.

Unlike previous temperate-climate or exercise-focused HRV studies, the present study highlights the integrated influence of circadian, age-related, and environmental factors on equine autonomic regulation. Circadian variation appeared to be driven more by activity-related modulation than by purely endogenous control, whereas aging was associated with reduced short-term vagal activity and decreased autonomic flexibility.

### Blunted circadian autonomic modulation under tropical conditions

Circadian patterns of HR and HRV in this study reflect a dynamic interaction between endogenous rhythms and daily management-related activities. The overall pattern indicates increased autonomic activation during daytime, followed by partial recovery during nighttime. However, the absence of a consistent nocturnal increase in parasympathetic-related indices, including RMSSD, pNN100, and HF, indicates that typical nocturnal vagal predominance was attenuated. Although long-term variability indices, including SDANN and VLF, appeared to increase during evening and nighttime periods, short-term vagal modulation remained relatively unchanged, showing that nocturnal slowing of HR was not accompanied by a proportional increase in beat-to-beat parasympathetic activity. Overall, these findings reflect a blunted circadian autonomic rhythm.

These findings differ from previous studies conducted under temperate conditions, where a clear nocturnal increase in parasympathetic activity and stronger circadian amplitude are typically observed [17, 24]. In contrast, the attenuated nocturnal pattern observed in this study suggests that environmental and management-related factors may interfere with normal autonomic recovery. In particular, daily management routines, including late-afternoon exercise followed by feeding, as outlined in the activity schedule (Table 2), may contribute to transient sympathetic activation extending into the early nighttime period. In addition, environmental conditions characteristic of tropical regions, such as minimal nocturnal cooling and high humidity, may limit physiological recovery during rest [25, 26]. Nocturnal disturbances, including exposure to blood-feeding insects, may further disrupt rest and reduce parasympathetic predominance [16]. However, because sleep quality and nocturnal disturbances were not directly assessed, these mechanisms remain speculative.

### Age-associated decline in vagal regulation

Across age groups, the findings provide evidence that aging is associated with decreased parasympathetic regulation and reduced overall autonomic flexibility. This was primarily reflected by declines in short-term HRV indices, including NN, SDNN, RMSSD, pNN100, and HF, together with an increased LF/HF ratio. In contrast, the less pronounced change in long-term variability indices, including SDANN and VLF, suggests that slower regulatory mechanisms may remain relatively intact with aging. Overall, these findings indicate reduced vagal balance or a shift toward sympathetic predominance in older horses.

The findings are consistent with previous studies in horses and humans, which reported an age-related decline in parasympathetic regulation [10, 18, 27–29]. In contrast, the relative stability of long-term HRV parameters observed in the present study may be explained by differences in their underlying physiological regulation. Human studies have shown that short-term indices tend to decline more rapidly with age, whereas long-term indices exhibit a more gradual, continuous decrease [30]. These long-term components are influenced by broader regulatory systems, including neurohumoral control, thermoregulation, and peripheral vasomotor tone, rather than solely by rapid parasympathetic modulation [31, 32]. As a result, long-term variability may appear relatively preserved compared with short-term vagal indices. Although direct evidence in horses remains limited, this framework may help explain the pattern observed in the present study. Importantly, the present findings extend previous equine studies by demonstrating that these age-related autonomic changes occur under tropical environmental conditions, where persistent heat exposure, high humidity, and other environmental stressors may further modulate autonomic regulation.

### Environmental modulation of cardiac autonomic function

Environmental factors were associated with cardiac autonomic regulation in horses, reflecting dynamic responses to environmental stressors. Overall, the observed patterns indicate that environmental stressors, particularly air pollution, may disrupt autonomic balance. Higher AQI and PM2.5 were consistently associated with reductions in several HRV indices, including SDNN, RMSSD, pNN100, and HF, together with an increased LF/HF ratio, demonstrating altered autonomic modulation.

Thermal stress is generally associated with increased sympathetic activity [33, 34], and high humidity can exacerbate heat load by impairing evaporative cooling [35, 36]. However, neither temperature nor feels-like temperature showed significant associations with HRV indices in the present study, likely because of the relatively narrow thermal range during the tropical winter period. In contrast, the negative association between humidity and HR observed in this study likely reflects day–night environmental patterns rather than a direct physiological effect of humidity itself, as humidity increased at night when ambient temperature and light intensity declined (Table 3). This interpretation is supported by the observed effects of light intensity, which showed associations consistent with increased arousal and sympathetic activation, leading to elevated HR and shortened NN [37, 38]. The accompanying reduction in SDANN likely reflects a decrease in long-term HRV rather than a specific shift between sympathetic activation and vagal withdrawal [39].

### Air pollution-associated autonomic imbalance

Air pollution, particularly PM2.5, was consistently associated with altered autonomic regulation, reflected by reductions in HRV indices alongside negative correlations with HR and respiratory cycle. These findings suggest a complex and potentially species-specific response in horses. Unlike the tachycardia commonly reported in humans and rodents [40–43], horses exhibited reductions in HR and respiratory rate, which may reflect a vagally mediated reflex response. Inhalation of PM2.5 may stimulate pulmonary irritant receptors and C-fibers, triggering reflex pathways that reduce both cardiac and respiratory activity [44, 45]. Despite this apparent vagal activation, the concurrent reduction in HRV indicates disruption of normal autonomic regulation rather than enhanced parasympathetic stability. This dissociation suggests that PM2.5 exposure may induce competing neural effects, including reflex vagal responses alongside impaired autonomic adaptability. In addition, PM2.5 is known to promote systemic inflammation and oxidative stress, which may further compromise autonomic control [40, 44].

Although the impact of PM2.5 on equine health is an emerging field with limited literature, existing studies have primarily documented localized respiratory effects, such as increased pulmonary inflammatory cells and impaired racehorse performance, even at low concentrations of 4–24 µg/m³ [46, 47]. Notably, the mean PM2.5 level in the present study was approximately 53 µg/m³, which was more than twofold higher than those reported in earlier equine research and nearly fourfold higher than the World Health Organization daily threshold of 15 µg/m³ [48]. This concentration is representative of the peak pollution season in Bangkok in January 2025, characterized by prolonged atmospheric stagnation and poor dispersion. This markedly higher exposure may account for the pronounced magnitude of autonomic alterations observed in the study horses. To our knowledge, this is among the first studies to directly examine the relationship between ambient PM2.5 levels and resting HRV parameters in horses under real-world conditions.

### Study limitations

This study has several limitations that should be considered. The sample size was relatively small (n = 5 per group), which may limit statistical power. Data were collected during a single seasonal period, the tropical winter in late January, and may not represent conditions across other seasons with greater heat stress. Although management conditions were similar, no direct measurements of physical activity, sleep, nocturnal disturbances such as insect exposure, ammonia, dust, or noise levels were obtained, which may have influenced circadian HRV patterns. HRV was assessed using a Polar H10 sensor rather than a gold-standard electrocardiographic system, and potential measurement limitations under field conditions cannot be excluded. However, the Polar H10 has been widely used in both human and animal HRV research and has demonstrated acceptable accuracy for RR interval detection, particularly under controlled or minimally active conditions [13, 49]. Given that the present study focused on HRV derived from RR intervals rather than detailed electrocardiographic morphology, the use of this device is considered appropriate for the study objectives. Finally, the study population consisted of riding-school horses in an urban tropical setting, which may restrict generalizability to other equine populations, management systems, or regions.

### Practical implications for equine welfare

The findings highlight that older horses may be more susceptible to environmental stressors and show clear signs of autonomic dysfunction. Because these changes may intensify under hotter conditions beyond the tropical winter, age-specific management strategies for older horses are warranted. Furthermore, the significant association between PM2.5 exposure and autonomic rigidity underscores air quality as an important welfare concern. Practical interventions, including avoiding exercise during high-pollution periods, reducing physical demands when air quality is poor, improving dust suppression, and monitoring air quality in urban stables, may help support the physiological stability and welfare of horses exposed to these environmental conditions.

### Future research directions

Future studies should include larger sample sizes and multi-seasonal data collection to better capture the full range of tropical environmental variability. In addition, continuous monitoring of activity, sleep, and environmental disturbances, including insect exposure and stable conditions, would improve the interpretation of circadian patterns. Finally, controlled experimental studies are needed to clarify causal relationships between air pollution and autonomic regulation in horses.

## CONCLUSION

This study demonstrated that horses maintained under tropical urban conditions exhibit attenuated circadian autonomic modulation, characterized by only modest nocturnal reductions in HR and the absence of a pronounced nocturnal increase in parasympathetic-related HRV indices. Age-related alterations in autonomic regulation were evident, with older horses showing significantly reduced vagal activity, lower HRV indices, and higher LF/HF ratios, indicating diminished autonomic flexibility and a shift toward sympathetic predominance. In addition, elevated PM2.5 and AQI levels were consistently associated with impaired autonomic balance, as reflected by reduced RMSSD, pNN100, HF power, and total power, along with an increased LF/HF ratio. These findings provide the first evidence linking ambient air pollution with alterations in resting HRV in horses under real-world tropical conditions.

A major strength of this study was the integrative approach combining continuous 24-hour HRV monitoring, age stratification, and synchronized real-time environmental measurements under field conditions. Unlike previous studies conducted primarily under temperate climates or focused on individual determinants of HRV, the present study simultaneously evaluated circadian rhythm, aging, and environmental influences, thereby providing ecologically relevant insights into equine autonomic regulation in tropical urban environments.

Overall, the findings indicate that environmental conditions and aging exert a greater influence on cardiac autonomic function than time of day alone in tropical horses. The observed blunting of nocturnal parasympathetic activity and the association between air pollution and autonomic dysfunction suggest that horses may exhibit a unique physiological response to chronic environmental stressors. These results emphasize the importance of age-specific management and environmental monitoring to safeguard equine welfare. Furthermore, the study provides a foundation for future investigations to elucidate the mechanisms underlying pollution-induced autonomic alterations and to develop mitigation strategies to promote the health and well-being of horses living in increasingly urbanized environments.

## DATA AVAILABILITY

The data generated during the study are included in the manuscript.

## GENERATIVE AI DECLARATION

The authors declare that generative artificial intelligence (AI) tools were used solely to improve language, grammar, and readability during manuscript preparation. All scientific content, data analysis, interpretation of results, and conclusions were developed and verified by the authors. The authors take full responsibility for the accuracy, integrity, and originality of the work presented, and no AI tool was listed as an author.

## AUTHORS’ CONTRIBUTIONS

AI: Conceptualization, methodology, investigation, data curation, visualization, and writing – original draft. PS and PP: Investigation. NS: Methodology. JP: Data curation, formal analysis, and visualization. WP: Methodology, writing – review and editing, supervision, and validation. KNL: Methodology, writing – review and editing, and formal analysis. PR: Methodology, supervision, and writing – review and editing. All authors have read and approved the final version of the manuscript.
